# Conflict-related environmental damages on health: lessons learned from the past wars and ongoing Russian invasion of Ukraine

**DOI:** 10.1265/ehpm.22-00122

**Published:** 2022-09-02

**Authors:** Kouji H. Harada, Sani Rachman Soleman, Jeremy Sea Meng Ang, Antoine P. Trzcinski

**Affiliations:** 1 Department of Health and Environmental Sciences, Kyoto University Graduate School of Medicine, Yoshida Konoe, Sakyo, Kyoto 606-8501, Japan; 2 Department of Public Health, Faculty of Medicine, Universitas Islam Indonesia, Yogyakarta, 55584, Indonesia; 3 Institute of Food Safety and Health, College of Public Health, National Taiwan University, 17, Xuzhou Rd, Zhongzheng District, Taipei City, 100, Taiwan; 4 School of Agriculture and Environmental Science, Faculty of Health, Engineering and Sciences, University of Southern Queensland, West Street, 4350 Queensland, Australia

**Keywords:** Conflicts, Environmental impacts, Health risks, Ukraine, COVID-19

## Abstract

On 24 February 2022, Russian military forces invaded Ukraine. The fighting has already caused unimaginable conditions and millions of people were forced to flee their homes. For decades, conflicts have been linked to environmental pollution, exposure to radioactivity and heavy metals as well as infectious diseases. The invasion may cause specific environmental risks, like the release of radioactive substances from nuclear power plants and contaminated soils. Because international collaboration is one of the most effective ways to address environmental problems, it is critical to establish scientific bodies within a global framework to identify concrete actions and tangible measures to provide immediate assistance to citizens. This commentary discusses the above issues from lessons learned from the past wars and the way forward in the Russian invasion of Ukraine.

## Introduction: Russian invasion of Ukraine

On 24 February 2022, Russian military forces invaded Ukraine. The fighting has already caused catastrophic conditions, millions of people are forced to escape from their homes, and thousands of them are trapped under the wreckage of buildings. Political, economic, and potentially military supports and responses have been debated, but the health consequences should be considered severe. Most evident are direct deaths by the conflict. There have been reported 4,633 civilian casualties, 1982 killed and 2651 injured including 147 children as of April 15, 2022 [1].

As Ukraine is an industrialized country (GDP composition: agriculture, 12.2%; industry, 28.6%; services, 60% in 2017 [2]), the invasion may cause specific environmental risks, like the release of radioactive substances from nuclear power plants. The most deleterious threat is spilling toxic waste from chemical facilities and the subsequent contamination of residential areas. In addition, it also may cause secondary pollution of food products. These will grow as fighting continues and could get a lot worse in the course of the conflict. This devastating conflict will cause huge human life threats such as food shortage, infectious diseases, and environmental contamination-induced health problems. Furthermore, the conflict will impact food security, clean water supply, energy policy, and greenhouse gas emission.

## Conflict-related environmental damage to health

For decades, conflicts have been linked to environmental pollution, exposure to radioactive elements [3] and heavy metals [4] as well as water-borne diseases [5, 6]. For instance, the war in Iraq (2003–2011) has cumulatively devastated soil, water and air [3]. The presence of radioactive substances in the soil such as uranium-238, uranium-235, cesium-137, and cobalt-60 has been documented in several cities in Iraq. Radioactive soil particles spread due to dust storms and contaminated vegetables, animal organs, and the food chain. Thus, radioactive elements enter the human body through inhalation or ingestion of contaminated food causing cancers and birth defects. Due to exposure to depleted uranium in Iraq, there was a higher risk for breast cancer compared to other types of cancers [3]. Cancer patients were found to have a higher average uranium concentration of 1.6 µg/L compared to healthy females with 1.0 µg/L. Furthermore, the number of congenital anomalies increased 17-fold in Al Basrah hospital compared to a decade before the conflict started. Lead concentration in a deciduous tooth of a child with congenital disorders was found to be three times higher (4.19 µg/g) than in children from unexposed areas [3].

Additionally, a major cholera outbreak was reported during the 2017 crisis in Yemen [6]. The World Health Organization (WHO) recorded more than 1 million cumulative cholera cases. The fatality rate was high, particularly among children. Scarcity of water and destruction of water and sanitation facilities due to bombing in early 2015 induced water-borne diseases. The Harvard Study Team recorded that as a consequence of the Persian Gulf Armed Conflict, children in some cities in Iraq are suffering from infectious diseases [7]. The study claimed that the destruction of facilities, clean water issues, and food contamination had led to disturbing long-term consequences for health, with an alarming increase in gastroenteritis, cholera, and typhoid fever. The government stated an epidemic surge in inpatient and overwhelming cases in outpatient. The highest prevalence of malnourished children in hospitals and health centers, in the form of marasmus and kwashiorkor, might be attributable to the epidemic level of cholera, gastroenteritis and typhoid fever. These conditions were aggravated by the loss of hospitals and healthcare centers, lack of diagnostic tools, and trained healthcare workers [7].

It should be noted that the impacts of conflicts will depend on the environmental and socio-economic situations in the region. The attack on a nuclear power plant has not yet been fully documented in terms of air, soil, and water contamination. COVID-19 pandemic is also specific to this conflict to affect the consequence. In the next section, we discuss the potential hazards of this conflict in Ukraine.

## What may happen and how can we prevent the damages?

As the Russian-Ukraine conflict continues, widespread destruction of military facilities, equipment, vehicles and civilian buildings can lead to air, water, and land contamination due to the release of chemical substances which can take very long to degrade naturally [8]. Smoke resulting from explosions consists of toxic gases and particulate matter which spreads over residential areas, and these pollutants eventually migrate to contaminate water and soil. Thus groundwater, agriculture, and animal products become a primary source of poisoning in humans. Regarding the Chornobyl nuclear power plant, the level of gamma radiation has been reported to be 28 times higher than the annual limit [9], which is likely due to the resuspension of dust from military vehicle mobilization. Pulverized materials resulting from explosive weapons encompassed asbestos, heavy metal, and combustible products. In 2017, the Ukrainian government announced a ban on asbestos but the implementation is ongoing, and the annual consumptions were 10,400–42,000 from 2012–2016 [10].

Explosives, propellants and degradation products on the other hand may include nitroaromatics such as 2,4,6-trinitrotoluene or TNT, nitroamines such as hexahydro-1,3,5-trinitro-1,3,5-triazine, nitrate esters and ammonium picrate [11]. Some aromatics molecules will not degrade readily if released accidentally into the environment and residues from detonation sites are usually found at µg/L levels in groundwater, but sometimes even at mg/L levels. These concentrations in drinking water will lead to acute and chronic toxicity according to the US Environmental Protection Agency [11]. Commercial explosives usually contain some form of ammonium nitrate which will eventually leach into water. Ammonium can lead to toxicity in fish and eutrophication, while nitrate can lead to methemoglobinemia in infants who drink the water. Detonation of explosives will lead to the release of CO_2_, N_2_, NH_3_, N_2_O, NO and NO_2_ into the atmosphere with some of these gases known to generate greenhouse effects [12].

Furthermore, the destruction of sewerage pipes and wastewater treatment facilities can lead to surface water, groundwater, and soil contamination by pathogens. An impact on energy resources and policies in the world as a long war goes on, may increase greenhouse gas emissions that impose climate change. Finally, the scope of environmental hazards includes damage not only to local, but also to global populations.

Environmental damage, pollution, and their negative impacts in this conflict need to be evaluated. Further research and development for effective humanitarian responses will mitigate health risks and prioritize reconstruction programs. Immediate action is necessary to support vulnerable populations from the impact of environmental destruction such as the following four issues:

The first is to ensure environmental safety from radioactive substances. It is imperative to note the accessibility to environmental and humanitarian aid to prevent releases of radioactive and chemical substances in Chornobyl and surrounding areas. As the Chornobyl power plant is still decommissioning, stopping the process could raise the risk of a major accident. Moreover, Zaporizhzhia nuclear power plant in southeast Ukraine was attacked on 4 March 2022 and suffered from a fire. While the release of radioactive materials was not confirmed [13], tight protection in the plant is warranted to prevent a devastating accident across the globe. The plant produces a massive amount of radioactive materials, which would be more hazardous than the Chornobyl accident in 1986. In case of an uncontrolled radioactive leak, a lack of rapid response, access, equipment and trained personnel may lead to a catastrophic scenario with global consequences. A collaborative action between non-governmental organizations (NGOs) would be beneficial to prevent potential environmental hazards and monitor land contamination. Having such a system in place would ensure a more efficient cleanup once the war ends.

The second is to maintain clean water and food supply. The depleted uranium from projectile wreckage will leave uranium dust in the environment. Thus, humans could be exposed through inhalation and ingestion by consuming contaminated food, vegetable, and meat products [3]. Accessibility and quality of water are other serious problems. Several water facilities were damaged in Ukraine. Russian forces deliberately destroyed a concrete dam providing water to the city of Kherson in Southern Ukraine [14]. In Mariupol, Russian soldiers shut off water supply completely leaving the entire population with no water or sanitation. Attacks on civilian water infrastructure violate international conventions, but Moscow has deliberately targeted water infrastructures to force key strongholds to surrender [15]. As a result, it is estimated that 1.4 million people now live without clean water [16]. The Geneva List of Principles on the Protection of Water Infrastructure aims to prevent the impact of military conflict on water facilities and resources, enhancing collaboration to protect these vital resources (https://www.genevawaterhub.org/resource/geneva-list-principles-protection-water-infrastructure). The principle also points out that water-related infrastructures are used by civilians and should therefore not be deliberately attacked. This draft pays attention to the increasing use of water infrastructure during the war and strengthens the role of water in peacebuilding concerns. An agreement to implement international efforts and humanitarian law concerning water is paramount to ensure health resilience for vulnerable groups.

The third is to evaluate the impact on energy resources and greenhouse gas emissions. Several Western countries restricted Russian fossil fuel imports and the Russian federation also cut off their supplies to the European Union (EU). This will cause changes in energy policies in the EU to decrease fossil fuel power generation. A recent analysis showed a long-term environmental benefit of reducing CO_2_ (6.6% in 2030) and air pollutant emissions such as fine particulate matter and sulfur dioxide [17]. On the other hand, the destruction of buildings and fires would generate greenhouse gasses as it has been in 1943 during the Hamburg bombing where it has been estimated that between 3.5 and 21 million t CO_2_ were emitted [18]. The overall amount in the current war remains unknown, but global monitoring using remote sensing will provide comprehensive estimates and predict the effects on climate change and health.

The fourth is to prevent emerging infectious diseases. Some infectious diseases have raised serious concerns in Ukraine, such as Polio, COVID-19, and cholera. Polio resumed in Ukraine because of the lowering rate of immunization. Polio is a severe threatening virus that can cause paralysis in children, and 20 cases have been confirmed in the Rivne and Zakarpattia regions in 2021 [19]. Unvaccinated children are at high risk of being exposed to the virus. In addition, daily COVID 19 cases surpassed 25,000 in February 2022 [20]. The situation is dire since almost half of the population is unvaccinated. The spread of COVID-19 would decrease the capacity of healthcare providers for other diseases [21] and the current war with Russia will reduce even more the number of beds available to treat COVID-19 patients. As the war goes on, the cholera outbreak may worsen in Ukraine because of lack of water sanitation, poor hygiene, and food contamination. These issues call for a collaborative humanitarian network to prevent catastrophic illnesses across Ukraine. Work with the Ukrainian government should be undertaken to ensure effective measures for implementing emerging infectious diseases response plans, particularly in the low-rate vaccination regions. The ultimate goal is to ensure populations are free from vaccine-preventable diseases [19, 22] and the provision of sustainable water, sanitation, and hygiene [23].

## Need for international collaboration to evaluate and prevent environmental health risks

Although this war has drawn global attention for its environmental health damage in the region, there has been little assistance from the international community to solve this issue. However, because international collaboration is one of the most effective ways to address environmental problems, it is critical to establish scientific bodies within a global framework to identify concrete actions and tangible measures to provide immediate assistance to citizens. For this reason, we suggest the implementation of monitoring and controlling processes which can be undertaken by international scientific and academic communities to tackle environmental health issues. International collaboration to evaluate and prevent environmental health risks is a way to support scientific evidence of the public health impacts of an armed conflict.

Competent laboratories are essential to monitor the environment from harmful chemical substances and inform the public and governments. To achieve this, the international community can provide appropriate infrastructure and data management tools to detect and monitor threats to public health. Improving health professionals’ education and training at the international level is also critical to address the shortage of health professionals after warfare. Secondly, controlling emerging infectious diseases has become a top priority in creating a safe environment for civilians to rebuild the country. Hence, the international organization should provide a surveillance tool–linking databases and sharing epidemic information. A comprehensive preventative and intervention approach should also be implemented with international cooperation in environmental protection. Effective international cooperation in environmental protection will require the active participation of international organizations and scientific communities. Not only research activities, but strong advocacy from the community for peace will also remain crucial as stated by the Japanese society for hygiene (http://www.nihon-eisei.org/seimei220407/).

Finally, Russia’s forces were not complying with international humanitarian law and continue their brutal attacks on civilian areas. The Ukrainian people are currently in danger. It is our responsibility to highlight the issues, at least, in environmental health.
